# Pediatric Nephrology in Primary Care: The Forest for the Trees

**DOI:** 10.3389/fpubh.2015.00227

**Published:** 2015-10-06

**Authors:** Donald E. Greydanus, Vimal Master Sankar Raj, Joav Merrick

**Affiliations:** ^1^Department of Pediatric and Adolescent Medicine, Western Michigan University Homer Stryker MD School of Medicine, Kalamazoo, MI, USA; ^2^Department of Pediatric Nephrology, Children’s Hospital of Illinois, Peoria, IL, USA; ^3^National Institute of Child Health and Human Development, Jerusalem, Israel; ^4^Office of the Medical Director, Health Services, Division for Intellectual and Developmental Disabilities, Ministry of Social Affairs and Social Services, Jerusalem, Israel

**Keywords:** pediatrics, nephrology, primary care, kidney, public health

## Introduction

The first book on pediatrics in the Western world was published in 1544 AD by Thomas Phaer (1510–1560) – a truly assiduous Renaissance man of sagacity – physician, lawyer, poet, philosopher, and father of English pediatrics (1). However, sustained interest in diseases of children has come over the past two centuries as attention before that was primarily, and certainly continues to be, with diseases of adults (2, 3). Prior to this, medical care for children was provided by midwives, families, and family friends (2). As progress was made in various organs of the human, knowledge about the kidneys gradually arose. Much information was gained in both adult and pediatric nephrology in the twentieth century. One concept for the primary care clinician to consider is where is the field of pediatric nephrology going and what is the role of primary care in this regard?

## Twenty-First Century: Future Directions

As we carefully cast our eyes though the keyhole of science, one can see more wondrous advances in nephrology as this twenty-first century unwinds based on the work of previous scholars (Table 1) (2, 4). More understanding will develop in the complex interactions of vitamin D and the kidney based on the 1973 synthesis of 1,25(OH)_2_ vitamin D (4–6). Rickets was identified in the first part of the twentieth century and research on this defective disorder of mineralization will reach further progress (4). Mutations in podocyte genes were shown in the twentieth century to lead to renal dysfunction from hereditary podocytopathies that has opened up an active vein of study in this century (4, 7). The twentieth century Human Genome Project (HGP) has energized intense inquisition on genetic causes of renal disease and genetic testing in renal disease in this century with work on total human exome capture, next-generation sequencing, gene regulatory networks (GRN), genome-wide association studies (GWAS), the role of micro-RNAs (miRNAs) in renal disease, genotype-based risk prediction, and individualized genetic treatments (4, 8–11).

**Table 1:** **Evolving twenty-first century progress in nephrology**.

AIDS nephropathy
Anti-inflammatory disease and treatments
Antecedents of adult renal disease
Continuous ambulatory peritoneal dialysis (CAPD) vs. APD in children
Diabetic nephropathy
Genetic and genomic techniques/genetics (single gene disorders)
Hypertension and the kidney
Ion channel mutations
Molecular and cell biology
Nephrotic syndrome
Others (see text)
Pediatric kidney transplantation and graft rejection
Podocytopathies
Proteomics
Rickets
Stem cell therapies
Tissue engineering and regenerative medicine
Vitamin D and the kidney (proximal tubule interactions and others)

Modern science will continue its hunt for understanding of renal disorders by looking at twenty-first century progress in cell biology, epigenetics, metabolomics, proteomics, pharmacogenomics, other “omics,” integrins, inflammasomes, autophagy, stem cells, biomarkers, and other examples of Research and Development (12–20). At the turn of the twenty-first century, research revealed that the study of proteomics was being used to classify defects in such renal conditions as congenital nephrotic syndrome (NS), renal hypomagnesia, and Alport syndrome variants. Ion channel mutations were identified in the early 1990s as etiologically implicated in various renal tubular syndromes, nephrogenic diabetes, and polycystic renal disease; this erudite exploration will continue (4).

Simultaneous cognizance of diabetes from Areteus of Capadocia (81–138 AD – “melting away of flesh into urine”) and renal physiology over the eons have lead to twentieth and now twenty-first century research on diabetic kidney disease that will advance (21). Cross-over investigations between the current and past centuries include exploration of the cause and management of hypertension and its connection to renal physiology as well as the impact of the twenty-first century epidemic on obesity (22). Another cross-over epidemic from the previous century to the present one that will continue is AIDS/HIV, including AIDS nephropathy that was first identified in the 1980s (23).

Continuous ambulatory peritoneal dialysis (CAPD) in children was identified in the 1970s and 1980s; its usefulness will be studied further now and well into this century – in relation to such issues as risk (i.e., peritonitis) and automated peritoneal dialysis (APD) (24). Challenges and benefits of pediatric renal transplantation will persevere to occupy researchers in pediatric and adult nephrology (25). This exigent endeavor includes protocols for minimal utilization of steroids and calcineurin inhibitors, improved anti-HLA antibodies diagnosis, and innovative methods to reduce early acute rejection and graft loss (25). Transplantation problems continue including donor organ shortage, graft failure, and many complications. Research in the twenty-first century is looking at cell-based tools that utilize tissue engineering and regenerative medicine to allow replacement of dysfunctional renal cells with normally functioning cells and subsequent normalization of renal activity (26).

Research will also advance issues that initiated the field of pediatric nephrology in the latter half of the twentieth century – including improved diagnosis and management of acute kidney injury (AKI), polycystic kidney disease, vesicoureteral reflux (VUR), and the NS (27). For example, work continues in AKI with reference to its early identification and its relation to hemodynamic resuscitation protocols. An algorithm has been developed to serve as a beneficial diagnostic tool for primary care clinicians with regard to early diagnosis and management of renal cystic disease. Studies are also examining current protocols in VUR diagnosis and management. After hundreds of years of dealing with the NS in medicine, NS remains a complex condition often subject to relapse and remission in most patients despite management in the twenty-first century (27).

## Primary Care: A Need for Insightful Information

The current and future shortage of pediatric nephrologists necessitates steady, rejuvenated information on the pediatric kidney for primary care clinicians as they care for the child and adolescent with renal and genitourinary dilemmas and disorders (28–30). As noted by a number of experts, this shortage of nephrologists, including pediatric nephrologists, will continue into the twenty-first century (28–30). In view of this shortage and the rapidly increasing knowledge in pediatric nephrology as well understanding indications for referral to pediatric nephrologists in the twenty-first century, useful information aimed at primary care clinicians in these areas becomes increasingly important.

It is easy for a non-nephrologist to become lost in the complexities of renal physiology, diagnosis, and treatment – i.e., to miss the forest for the trees. In this expression, one can see so many trees but not understand they are part of a vast forest and fail to appreciate the forest itself.

As the primary care clinician consider the many details of pediatric nephrology (the “trees”) one can fail to appreciate basic concepts that they can manage (the “forest”) – as for example – effectively managing potentially benign but common conditions, such as transient proteinuria, orthostatic proteinuria, and microscopic hematuria without proteinuria. If one appreciates that the majority of kidney disease in children relates to hypertension, the primary care physician (PCP) can be energized to persistently and carefully monitor the blood pressure of all of his/her pediatric patients. Early diagnosis of hypertension and careful management of all these patients with the help of consultants is a vital and rewarding task of primary care clinicians and will prevent much renal disease in these pediatric patients. Management of dehydration is also a critical role of clinicians (31). It is important for the PCP to remain familiar with common scenarios in nephrology through conferences and workshops. An interest in this field is important as PCPs will continue to monitor many of these children as the shortage of pediatric nephrologists will continue. The primary care clinician who clearly sees this forest will be of immense help to their pediatric patients and not miss the forest for the trees in pediatric nephrology.

I’m always astonished by a forest. It makes me realize that the fantasy of nature is much larger than my own fantasy. I still have things to learn.

Gunter Grass (1927–2015).

## Conflict of Interest Statement

The authors declare that the research was conducted in the absence of any commercial or financial relationships that could be construed as a potential conflict of interest.
